# GPs’ awareness of car driving among oldest patients: exploratory results from a primary care cohort

**DOI:** 10.3399/BJGPO.2020.0145

**Published:** 2021-03-24

**Authors:** Verena Leve, Michael Pentzek, Angela Fuchs, Horst Bickel, Dagmar Weeg, Siegfried Weyerer, Jochen Werle, Hans-Helmut König, André Hajek, Dagmar Lühmann, Hendrik van den Bussche, Birgitt Wiese, Anke Oey, Kathrin Heser, Michael Wagner, Melanie Luppa, Susanne Röhr, Wolfgang Maier, Martin Scherer, Hanna Kaduszkiewicz, Steffi G Riedel-Heller, for the AgeCoDe/AgeQualiDe Study Group

**Affiliations:** 1 Institute of General Practice (ifam), Medical Faculty, Heinrich Heine University Düsseldorf, Germany; 2 Department of Psychiatry and Psychotherapy, Klinikum rechts der Isar, Technical University of Munich, Germany; 3 Central Institute of Mental Health, Medial Faculty Mannheim/Heidelberg University, Mannheim, Germany; 4 Department of Health Economics and Health Services Research, Hamburg Center for Health Economics, University Medical Center Hamburg-Eppendorf, Germany; 5 Department of General Practice/Primary Care, University Medical Center Hamburg-Eppendorf, Germany; 6 WG Medical Statistics and IT-Infrastructure, Institute of General Practice, Hannover Medical School (MHH), Hannover, Germany; 7 Department of Neurodegenerative Diseases and Geriatric Psychiatry, University Hospital Bonn, Germany; 8 DZNE, German Center for Neurodegenerative Diseases, Bonn, Germany; 9 Institute of Social Medicine, Occupational Medicine and Public Health (ISAP), Medical Faculty, University of Leipzig, Germany; 10 Institute of General Practice, Faculty of Medicine, Kiel University, Kiel, Germany

**Keywords:** general practice, automobile driving, aged, 80 and over, physician—patient relations, validity, clinical decision-making

## Abstract

**Background:**

Increasingly more very old people are active drivers. Sensory, motor and cognitive limitations, and medication can increase safety risks. Timely attention to driving safety in the patient–doctor relationship can promote patient-centred solutions.

**Aim:**

To explore the following questions: do GPs know which patients drive a car? Is fitness to drive addressed with patients?

**Design & setting:**

Cross-sectional data from patient interviews and GP survey in the ninth follow-up phase of a prospective primary care cohort (the German Study on Ageing, Cognition and Dementia in Primary Care Patients (AgeCoDe) and the Study on Needs, Health Service Use, Costs and Health-Related Quality of Life in a large sample of ‘oldest-old’ primary care patients (≥85 years; AgeQualiDe)) .

**Method:**

The sample consisted of patients in the age group ≥85 years and their GPs. Independent reports were gathered on driving activity from the GP and the patient, and information was gained from GPs on whether driving ability was discussed with the patient. Statistical analyses included validity parameters and bivariate characterisation of subgroups (non-parametric significance tests, effect size).

**Results:**

Self-reports of 553 patients were available (69.5% female; mean age 90.5 years; 15.9% drive a car). For 427 patients, GP data were also available: GPs recognised 67.1% correctly as drivers and 94.9% as non-drivers. GPs said that they had discussed fitness to drive with 32.1% of potentially driving patients. Among drivers who were not recognised and with whom driving had not been discussed, there were more patients with a low educational level.

**Conclusion:**

The GP’s assessment of driving activity among very old patients showed moderate sensitivity and good specificity. Driving ability was seldom discussed. Asking an appropriate question during assessment could increase GPs’ awareness of older patients’ automobility.

## How this fits in

In Germany, there are no statutory driver safety checks in general practice. Driving has high individual relevance for older people. The results show that GPs are moderately aware of driving among the oldest patients, but seldom address it with patients. A standardised question within the geriatric assessment might enhance GPs’ awareness and communication.

## Introduction

In Europe, individual transport has gained significantly in importance over the past years.^1^ In 2018, travel by car reached 83% of all passenger transport in EU27 member states. For older people, driving is essential for maintaining their individual mobility and autonomy, especially when access to social participation and infrastructure in rural or suburban areas is restricted.^2–4^

Owing to the trends of the growing proportion of older and very old people in the population in Europe,^5^ an increase in the number of older drivers can also be expected.^1–4^ Some European countries, for example, the Netherlands, Ireland, Finland, and Denmark, require driving licence renewal including medical check-ups of fitness to drive based on age limits.^6^ The majority of European countries rely on the responsibility of drivers and self-regulated driving cessation. In Germany, driving licences are valid for an unlimited period and can only be withdrawn by the responsible authority.^6,7^ In Germany, GPs are not obliged to assess medical fitness to drive or to report limited driving ability to licensing authorities.^6,8^ As a matter of principle, a driver is responsible for participating in road traffic only if there are no relevant restrictions on driving ability and other road users are not endangered. However, with increasing multimorbidity, higher age can be associated with reduced driving safety; for example, cognitive limitations can influence reaction times and attention, and physical limitations can affect agility or audio-visual capabilities.^9,10^ The most common diseases considered to pose risks to driving safety include cardiovascular diseases, dementia, hypertension, diabetes, mental illness, and degenerative joint diseases.^9,10^ GPs play a major role in the care of patients with multimorbidity and addressing (basic and instrumental) activities of daily living, including mobility issues. Older patients frequently visit general practice and GPs are important contact persons in health and psychosocial matters.^11^ Although the issue of road safety of older drivers is an established research field,^2^ little is known about the factors that influence how driving safety issues are addressed with very old patients in general practice in Germany. In this article, the role of GPs in Germany is examined in addressing the issue of driving and recognising risks relevant to driving safety.

The following questions have been answered: (1a) do GPs know which of their very old patients drive a car? (1b) What is the difference between the groups of correctly recognised and overlooked drivers? (2a) Do GPs discuss fitness to drive with their patients? (2b) What is the difference between the groups of patients with whom the GP has spoken about fitness to drive, and those with whom they have not?

## Method

### Sample

The data presented here were collected within the framework of the studies AgeCoDe and AgeQualiDe. These studies focused on risk factors and diagnosis of dementia and health care of older adults in general. Details of these studies are presented in several publications.^12,13^ In six study centres (Bonn, Düsseldorf, Hamburg, Leipzig, Mannheim, Munich), 3327 patients from a total of 138 GP practices participated in the baseline study in 2003–2004, based on a random selection of 6619 GP patients. Inclusion criteria were: aged ≥75 years; no dementia diagnosis; and at least one contact with the GP in the past year. Exclusion criteria were: lack of willingness to give consent; insufficient German language skills; not being a regular patient at the practice; sole contact via home visits; living in an old people’s home or nursing home; serious illness that would lead to death within the next 3 months, as assessed by the GP; blindness; and deafness. Patients were assessed by trained scientific interviewers at home in a structured interview including psychometric tests. Independent from this, the GPs of the participating patients provided information, for example about patients’ diseases. Assessments were repeated in intervals of 1.5 years until 2017. Major drop-out reasons in the course of the study were refusal and death. Details on recruitment and participation in the course of the study are published elsewhere.^14^ As there is a lack of research about it, the topic of driving was explored in the last wave of the survey 2016–2017 (*n* = 643), only in patients with a Mini Mental Status Examination (MMSE) score^15^ of at least 19.

### Measures of driving activity from the perspective of patients and GPs

Patients were asked by the interviewers about their driving behaviour: 'Do you still drive a car?' (yes or no); if the answer was positive, questions on frequency and distance followed: 'How often do you drive?' (less than once a week, once a week, several times a week, or every day); 'Do you still drive ... ?' ('... only short distances that are necessary [for example, only to the nearest supermarket]' versus ' ... even longer distances [>15 minutes]').

Independently, the GP was asked to rate the patient’s current driving activity: 'Does the patient currently drive a car?' (yes, no, or do not know); in the case of a yes answer, an additional question was asked: 'Have you discussed the topic of fitness to drive with the patient?' (yes or no).

### Other variables

With regard to sociodemographic variables, age, sex, living situation (alone versus not alone), and educational status were considered, the latter operationalised according to the Comparative Analysis of Social Mobility in Industrial Nations (CASMIN) classification system (low, medium, high).^16^ Social network was recorded with the 6-item version of the Lubben Social Network Scale (LSNS;^17^ score 0–30, higher score = larger social network).

With regard to physical and clinical characteristics, patients were asked about problems with hearing, walking, and seeing (analysed dichotomously). The 15-item version of the Geriatric Depression Scale (GDS)^18^ was used to measure depressive symptoms (score 0–15, higher score = more depressive symptoms). Global cognitive performance was measured with the MMSE^15^ (score 0–30, higher scores = better performance). The presence of dementia was investigated with a comprehensive interview and test inventory,^19^ which combines international dementia criteria in a diagnostic algorithm. This includes testing of cognitive performance, assessment of activities of daily living, and informant reports in case of suspected dementia. Any medical indication of dementia was discussed in consensus conferences with the interviewers and experienced geriatricians or psychiatrists. The medication of patients was assessed directly at the patient’s home. For the purpose of this analysis, all anatomical therapeutic chemical (ATC) codes were determined and compared with the ATC codes on the DRUID list (EU project DRUID: 'Driving under the Influence of Drugs, Alcohol and Medicines').^20^ It was evaluated whether or not a patient was taking at least one drug in DRUID categories II and III, which are considered to be relevant to driving safety.

Driving behaviour was specified by patients in terms of frequency and distances (as above). As the only independent variable, existing diseases were not recorded at the patient’s home but from the GP (per list of 35 diseases). For the present analyses, the presence of one or more diseases relevant to driving according to the Driving Licence Ordinance^7^ was derived from this.

### Statistical analyses

For question (1a), the validity parameters sensitivity, specificity, and positive and negative predictive value were calculated (reference ‘patient information on driving’) as well as Cohen’s concordance measure ƙ. For question (2a), the percentages of patients with whom the GP discussed or did not discuss fitness to drive are shown.

The analyses for questions (1b) and (2b) were concerned with the bivariate characterisation of two samples (recognised versus unrecognised drivers, and patients with whom the family doctor discussed fitness to drive versus those with whom they did not discuss fitness to drive). Owing to small sample sizes and skewed distributions, non-parametric tests were used here: Mann–Whitney U-tests for continuous and ordinally scaled variables; and χ^2^ tests for categorical variables. Since the sample size was not planned a priori to answer the mentioned questions (exploratory analysis of an existing cohort), effect sizes were given in addition to *P* values.^21^ This served to ensure that relevant differences were not overlooked, even if they were not statistically robust in the given small sample. To calculate effect sizes, test parameters were converted into Cohen’s *d* (up to 0.19 no effect, 0.20–0.49 small effect, 0.50–0.79 medium effect, from 0.80 large effect); medium and large effects were interpreted.^22–24^ Analyses were performed with SPSS (version 25.0).

## Results

### (1a) Do GPs know which of their very old patients drive a car?

From 553 patients (69.5% female; mean age 90.5±2.7 years, 86–101 years) self-reports on driving activity were available: 15.9% drive a car. For 574 patients, a GP rating on driving activity was available (85.9% 'no, does not drive', 9.8% 'yes, drives', 4.4% 'don't know'). For 427 patients, both patient and GP data were available.

See Table 1 for the validity of the GP rating of driving activity.

**Table 1.** Validity of the GP rating of driving activity

		**Patient report** (reference):*'Do you drive a car?'*		**Total**	**Predictive values** (95% CI)
		*Yes*	*No*		
**GP rating:** *'Does the patient currently drive a car?'*	*Yes*	**49**	**18** ^a^	*n* = 67	PPV 73.1(63.1 to 81.3)
	*No*	**24** ^b^	**336**	*n* = 360	NPV 93.3(91.5 to 94.9)
**Total**		*n* = 73	*n* = 354	*n* = 427	
**Test accuracy** (95% CI):		Sensitivity 67.1 (57.9 to 74.6)	Specificity 94.9 (93.0 to 96.5)		**Concordance:**Cohen’s ƙ = 0.64 (95% CI 0.53 to 0.74)

^a^Including 15 x GP rating 'don't know'. ^b^Including 8 x GP rating 'don't know'. CI = confidence interval. PPV = positive predictive value. NPV = negative predictive value.

### (1b) What is the difference between the groups of correctly recognised and overlooked drivers?

Compared with detected drivers, overlooked drivers were more likely to have a low educational status (32.7% versus 54.2%, *P* = 0.200; Cohen’s *d* = 0.43). Recognised drivers drive longer distances (73.5% versus 45.8%; *P* = 0.021; *d* = 0.56), and also drive more frequently (*P* = 0.001; *d* = 0.72) than overlooked drivers. Overlooked drivers also had higher depression scores (*P* = 0.010; *d* = 0.62). Apart from group comparisons, it is important to note that 20.8% and 29.2% of overlooked drivers had a medication or disease relevant to driving safety, respectively. For details, see supplementary Table S1.

### (2a) Do GPs discuss fitness to drive with their patients?

In *n* = 81 cases, GPs rated patients as active drivers or patients whose driving status they did not know (the latter considered as 'fitness to drive not addressed with patient'). In *n* = 55 cases (67.9%) fitness to drive was not addressed with patients; in *n* = 26 cases (32.1%), the GP spoke with the patient about fitness to drive.

### (2b) What is the difference between the groups of patients with whom the GP has spoken about fitness to drive and those with whom they have not?

Patients with whom the GP discussed fitness to drive were somewhat older (*P* = 0.018; *d* = 0.56) and had a higher educational level than those with whom fitness to drive was not discussed (23.1% versus 49.1%; *P* = 0.074; *d* = 0.53). For details, see supplementary Table S2.

## Discussion

### Summary

The GP rating of driving activity in very old patients is substantially consistent with the patient report,^25^ with moderate sensitivity and good specificity. Approximately one-third of drivers are overlooked more frequently those with low driving activity. A higher proportion of overlooked drivers have a low educational status, as do those drivers with whom the GP does not discuss fitness to drive. The majority of patients with dementia are correctly rated by their GP as not (no longer) driving. Fitness to drive is only discussed with one-third of potentially driving AgeQualiDe patients.

### Strengths and limitations

The results refer to a primarily urban sample of above-average healthy and active very old patients with a high driving prevalence. Drivers are relatively seldom cognitively impaired, as has been discussed elsewhere.^26^ This is a highly selected sample of surviving and long-term motivated participants in a 13-year cohort study; corresponding biases cannot be excluded.^27^

Regarding the reported diagnostic parameters, it has to be considered that these are derived from an exploratory question within a cohort study. This is not a diagnostic accuracy study according to STARD criteria.^28^ Furthermore, these are the results of explorative bivariate analyses; multivariable models of independent influences of individual factors cannot be derived owing to small sample size. Multilevel analyses on the influence of GP characteristics on identifying and addressing driving are also not possible. Further research is needed here.

### Comparison with existing literature

Various studies show that for patients with a low socioeconomic and especially lower educational status more time in consultations is spent for physical examination and less time for communication on health behaviour and activities of daily living.^29,30^ A higher socioeconomic status is accompanied by a more active communication behaviour of patients. Patients control the conversation more strongly through questions and additional information, and doctors tend to provide more detailed information in this context.^29,30^ In the context of addressing issues of driving safety, this may mean that less information is exchanged beyond the acute cause of treatment with patients who have a lower educational status and a more passive communication behaviour. Thus, GPs may receive less information about daily activities and mobility behaviour. In addition, overlooked drivers tend to drive less frequently and for shorter distances. It can be assumed that these are routine journeys, for example, for shopping, visits to the doctor, and regular leisure activities in the closer environment,^2,3^ which patients may report less often to their GP. More frequent and longer distance trips that deviate from daily routines, such as leisure activities in a wider environment or vacation, may be more likely to be a topic of conversation in the consultation.

For AgeQualiDe patients, depression levels are significantly higher among overlooked drivers, even if they are not clinically relevant. This effect can be influenced by the interaction of various factors such as sex and living situation. Older women are more often affected by depression than men,^31^ as are single women.^32^ The proportion of these two groups is higher among overlooked drivers in the sample.

The majority of patients with dementia were correctly assessed by their GP as not (no longer) driving. Cognitive as well as visual impairment, old age, and limited health can promote self-regulated driving cessation.^33,34^

With only one-third of all potentially driving AgeQualiDe patients, the GP addressed fitness to drive. GPs frequently perceive this topic as difficult. Even in countries where GPs are in charge of assessing medical fitness to drive, GPs are concerned that addressing driving ability can be threatening to the patient–doctor relationship, and there are uncertainties in assessment.^35,36^ For example, a survey of Irish GPs showed that 71% basically felt responsible for addressing driving safety.^37^ When assessing medical factors such as physical limitations or effects of medication, GPs felt confident, but when assessing basic driving competence there were uncertainties and a lack of assessment tools for use in general practice.^35–38^ Also in a study with physicians in Switzerland, Sebo found that practice assessment tools, such as a self-administered pre-consultation questionnaire, would be useful in GP practice.^39^ At the same time, existing information offered by driving authorities is rarely used.^39^

A further reason for the low level of attention paid to the topic in general practice could also be a frequently reported ethical conflict. This arises from GPs' double aspiration of maintaining the mobility of older patients and reducing risk for other road users.^37,38,40,41^ One of the authors' focus group studies, which explored physicians' attitudes when addressing issues of driving safety in consultation with people with dementia in general practice in Germany, revealed that GPs refuse to be involved in mandatory assessing and reporting medical fitness to drive. Nevertheless, they see their role in patient-centred care in addressing driving safety issues with patients.^42^ Uncertainties regarding the legal basis of the reporting process, and also regarding liability issues, are seen as further barriers to addressing fitness to drive.^38,40^

### Implications for research and practice

According to the German Federal Statistical Office, older drivers are generally less likely to be involved in road accidents, especially compared with young drivers. Nevertheless, the risk of being the main perpetrator of accidents increases among very old drivers.^43^ Redelmeier *et al* ^41^ showed that the risk of accidents could be reduced if patients who are medically unfit to drive were contacted at an early stage. Against this background, early advice on driving safety risks for medical reasons is essential for maintaining mobility through the autonomous arrangement of mobility alternatives together with patients.

A standardised question on driving, which is integrated into the geriatric assessment, could increase GPs' knowledge about car driving of older patients and, above all, provide the impetus for a further discussion. Owing to the possible negative consequences and individual risks of driving cessation, which are associated with risks for social participation in general,^41,44,45^ addressing driving issues in general practice should be embedded in a trustful patient—doctor relationship and in an appropriate communicative strategy. Further research is needed to develop assessment tools for general practice settings that also consider national programmes on supporting driving safety, traffic, and licensing regulation, as well as responsibilities in assessing medical fitness to drive.

Driving is an important prerequisite for social participation, especially for older people in rural and suburban areas.^3^ To reduce the individual risks of driving cessation, a deeper understanding of not only utilitarian mobility needs, but also affective and aesthetic mobility needs of older people is needed.^3^ Addressing mobility behaviour in general practice at an early stage thus provides access to address patient-centred mobility alternatives to secure autonomy and enable social participation.

## References

[1] Eurostat (2020). People on the move. Statistic on mobility in Europe. https://ec.europa.eu/eurostat/cache/digpub/eumove/index.html?lang=en.

[2] Haustein S (2012). Mobility behavior of the elderly: an attitude-based segmentation approach for a heterogeneous target group. Transportation.

[3] Musselwhite C, Haddad H (2018). Older people’s travel and mobility needs: a reflection of a hierarchical model 10 years on. Qual Ageing Older Adults.

[4] Rudinger G, Donaghy K, Poppelreuter S (2006). Societal trends, mobility behaviour and sustainable transport in Europe and North America. European Journal of Transport and Infrastructure Research.

[5] Eurostat (2019). Ageing Europe. Looking at the lives of older people in the EU. https://ec.europa.eu/eurostat/documents/3217494/10166544/KS-02-19%E2%80%91681-EN-N.pdf/c701972f-6b4e-b432-57d2-91898ca94893.

[6] Siren A, Haustein S (2015). Driving licences and medical screening in old age: review of literature and European licensing policies. J Transp Health.

[7] Bundesministerium für Verkehr, Bau und Stadtentwicklung [Federal Ministry of Transport, Building and Urban Affairs] [Ordinance on the Admission of Persons to Road Traffic - Driver's License Ordinance of December 13, 2010 (BGBl. I p. 1980), last amended by Article 4 of the Ordinance of April 20, 2020 (BGBl. I p. 814)] *Verordnung über die Zulassung von Personen zum*
*Straßenverkehr – Fahrerlaubnis-Verordnung vom 13. Dezember 2010 (BGBl. I S. 1980), zuletzt durch Artikel 4 der Verordnung vom 20. April 2020 (BGBl. I S. 814) geändert.* (in German). https://www.gesetze-im-internet.de/fev_2010/index.html.

[8] Peitz J (2015). [Protection obligation of treating physicians and psychologists - a critical review of the risk and liability community] *Schutzpflichten behandelnder Ärzte und Psychologen - Eine kritische Betrachtung der Gefahren- und Haftungsgemeinschaft* (in German). Blutalkohol.

[9] Gräcmann N, Albrecht M [Assessment guidelines for fitness to drive. Reports of the Federal Highway Research Institute. Mensch und Sicherheit Heft M 115] *Begutachtungsleitlinien zur Kraftfahreignung BASt 2014; M115* (in German). https://www.bast.de/BASt_2017/DE/Verkehrssicherheit/Fachthemen/U1-BLL/Begutachtungsleitlinien.pdf?__blob=publicationFile&v=20.

[10] Henning J (2007). [Road safety advice for older road users - Handbook for clinicians. Reports of the Federal Highway Research Institute. Mensch und Sicherheit Heft M 189] Verkehrssicherheitsberatung älterer VerkehrsteilnehmerBASt 2007; M 189 (in German).

[11] Robert Koch Institut (RKI) (2015). [Health in Germany. Federal Health Reporting] *Gesundheit in Deutschland. Gesundheitsberichterstattung des Bundes 2015* (in German). https://www.rki.de/DE/Content/Gesundheitsmonitoring/Gesundheitsberichterstattung/GesInDtld/GesInDtld_node.html.

[12] Buczak-Stec E, Hajek A, van den Bussche H (2020). Frequent attendance in primary care in the oldest old: evidence from the AgeCoDe-AgeQualiDe study. Aging Clin Exp Res.

[13] Pentzek M, Wagner M, Abholz H-H (2019). The value of the GP's clinical judgement in predicting dementia: a multicentre prospective cohort study among patients in general practice. Br J Gen Pract.

[14] Eisele M, Kaduszkiewicz H, König H-H (2015). Determinants of health-related quality of life in older primary care patients: results of the longitudinal observational AgeCoDe study. Br J Gen Pract.

[15] Folstein MF, Folstein SE, McHugh PR (1975). "Mini-mental state". A practical method for grading the cognitive state of patients for the clinician. J Psychiatr Res.

[16] Brauns H, Steinmann S (1999). Educational reform in France, West-Germany and the United Kingdom: updating the CASMIN educational classification. ZUMA Nachrichten.

[17] Lubben J, Blozik E, Gillmann G (2006). Performance of an abbreviated version of the Lubben Social Network Scale among three European community-dwelling older adult populations. Gerontologist.

[18] Yesavage JA, Sheikh JI (1986). 9/Geriatric Depression Scale (GDS): recent evidence and development of a shorter version. Clin Gerontol.

[19] Zaudig M, Hiller W (1995). [SIDAM manual: Structured interview for the diagnosis of dementia of the Alzheimer type, multi-infarct (or vascular) dementia, and dementias of other etiologies according to DSM-III-R, DSM-IV and ICD-10] SIDAM-Handbuch: Strukturiertes Interview für die Diagnose einer Demenz vom Alzheimer Typ, der Multi-Infarkt- (oder vaskulären) Demenz und Demenzen anderer Ätiologie nach DSM-III-R, DSM-IV und ICD-10 (in German).

[20] Ravera S, Monteiro SP, de Gier JJ (2012). A European approach to categorizing medicines for fitness to drive: outcomes of the DRUID project. Br J Clin Pharmacol.

[21] Lenhard W, Lenhard A (2016). Berechnung von Effektstärken. https://www.psychometrica.de/effektstaerke.html.

[22] Cohen J (1988). Statistical Power Analysis for the Behavioral Sciences.

[23] Fritz CO, Morris PE, Richler JJ (2012). Effect size estimates: current use, calculations, and interpretation. J Exp Psychol Gen.

[24] Elis P (2010). The Essential Guide to Effect Sizes: Statistical Power, Meta-Analysis, and the Interpretation of Research Results.

[25] Landis JR, Koch GG (1977). The measurement of observer agreement for categorical data. Biometrics.

[26] Hajek A, Brettschneider C, Eisele M (2019). Prevalence and determinants of driving habits in the oldest old: results of the multicenter prospective AgeCoDe-AgeQualiDe study. Arch Gerontol Geriatr.

[27] Lewin A, Brondeel R, Benmarhnia T (2018). Attrition bias related to missing outcome data: a longitudinal simulation study. Epidemiology.

[28] Bossuyt PM, Reitsma JB, Bruns DE (2015). Stard 2015: an updated list of essential items for reporting diagnostic accuracy studies. BMJ.

[29] Verlinde E, De Laender N, De Maesschalck S (2012). The social gradient in doctor—patient communication. Int J Equity Health.

[30] Willems S, De Maesschalck S, Deveugele M (2005). Socio-economic status of the patient and doctor—patient communication: does it make a difference?. Patient Educ Couns.

[31] Kuehner C (2003). Gender differences in unipolar depression: an update of epidemiological findings and possible explanations. Acta Psychiatr Scand.

[32] Jacob L, Haro JM, Koyanagi A (2019). Relationship between living alone and common mental disorders in the 1993, 2000 and 2007 National Psychiatric Morbidity Surveys. PLoS One.

[33] Anstey KJ, Windsor TD, Luszcz MA, Andrews GR (2006). Predicting driving cessation over 5 years in older adults: psychological well-being and cognitive competence are stronger predictors than physical health. J Am Geriatr Soc.

[34] Bergen G, West BA, Luo F (2017). How do older adult drivers self-regulate? Characteristics of self-regulation classes defined by latent class analysis. J Safety Res.

[35] Jones K, Rouse-Watson S, Beveridge A (2012). Fitness to drive — GP perspectives of assessing older and functionally impaired patients. Aust Fam Physician.

[36] Sims J, Rouse-Watson S, Schattner P (2012). To drive or not to drive: assessment dilemmas for GPs. Int J Family Med.

[37] Kahvedžić A, McFadden R, Cummins G (2015). General practitioner attitudes and practices in medical fitness to drive in Ireland. J Transp Health.

[38] Brooks JO, Dickerson A, Crisler MC (2011). Physician knowledge, assessment, and reporting of older driver fitness. Occup Ther Health Care.

[39] Sebo P (2020). Physicians' views on the usefulness of practical tools for assessing the driving ability of older drivers: a cross-sectional study. Fam Med Community Health.

[40] Brubacher J, Renschler C, Gomez AM (2018). Reporting unfit drivers: knowledge, attitudes and practice of BC physicians. University of British Columbia. http://med-fom-emerg.sites.olt.ubc.ca/files/2018/03/Fitness-to-Drive-Survey-final-2018-03-06.pdf.

[41] Redelmeier DA, Yarnell CJ, Tibshirani RJ (2013). Physicians' warnings for unfit drivers and risk of road crashes. N Engl J Med.

[42] Leve V, Ilse K, Ufert M (2017). Autofahren und Demenz. Z Gerontol Geriat.

[43] German Federal Statistical Office (destatis) (2019). [Traffic accidents: Road accidents involving seniors 2018] *Verkehrsunfälle: Unfälle von Senioren im Straßenverkehr 2018* (in German). https://www.destatis.de/DE/Themen/Gesellschaft-Umwelt/Verkehrsunfaelle/Publikationen/Downloads-Verkehrsunfaelle/unfaelle-senioren-5462409187004.pdf?__blob=publicationFile.

[44] Marottoli RA, de Leon CFM, Glass TA (2000). Consequences of driving cessation: decreased out-of-home activity levels. J Gerontol B Psychol Sci Soc Sci.

[45] Musselwhite CBA, Shergold I (2013). Examining the process of driving cessation in later life. Eur J Ageing.

